# Combined pembrolizumab and bevacizumab therapy effectively inhibits non-small-cell lung cancer growth and prevents postoperative recurrence and metastasis in humanized mouse model

**DOI:** 10.1007/s00262-022-03318-x

**Published:** 2022-11-10

**Authors:** Tianyun Qiao, Jinbo Zhao, Xiangbing Xin, Yanlu Xiong, Wenwen Guo, Fancheng Meng, Hui Li, Yangbo Feng, Hui Xu, Changhong Shi, Yong Han

**Affiliations:** 1 Department of Thoracic Surgery, Air Force Specialty Medical Center, Fourth Military Medical University, Xi’an, 710032 China; 2 Department of Thoracic Surgery, Tangdu Hospital, Fourth Military Medical University, Xi’an, 710032 China; 3 Laboratory Animal Center, Fourth Military Medical University, Xi’an, 710032 China; 4 School of Basic Medicine, Fourth Military Medical University, Xi’an, 710032 China

**Keywords:** Anti-programmed death 1, Anti-vascular endothelial growth factor, Humanized mouse model, Immunotherapy, Neoadjuvant therapy, Non-small cell lung cancer

## Abstract

Antibodies targeting the programmed cell death protein 1/programmed cell death ligand-1 (PD-1/PD-L1) pathway have dramatically changed the treatment landscape of advanced non-small cell lung cancer (NSCLC). However, combination approaches are required to extend this benefit beyond a subset of patients. In addition, it is of equal interest whether these combination therapy can be applied to neoadjuvant therapy of early-stage NSCLC. In this study, we hypothesized that combining immunotherapy with anti-angiogenic therapy may have a synergistic effect in local tumor control and neoadjuvant therapy. To this end, the effect of combination of bevacizumab and pembrolizumab in humanized mouse models was evaluated. Furthermore, we innovatively constructed a neoadjuvant mouse model that can simulate postoperative recurrence and metastasis of NSCLC to perform neoadjuvant study. Tumor growth and changes in the tumor vasculature, along with the frequency and phenotype of tumor-infiltrating lymphocytes, were examined. Additionally, in vivo imaging system (IVIS) was used to observe the effect of neoadjuvant therapy. Results showed that combination therapy could inhibited tumor growth by transforming tumor with low immunoreactivity into inflamed (‘hot’) tumor, as demonstrated by increased CD8^+^granzyme B^+^ cytotoxic T cell infiltration. Subsequent studies revealed that this process is mediated by vascular normalization and endothelial cell activation. IVIS results showed that neoadjuvant therapy can effectively prevent postoperative recurrence and metastasis. Taken together, these preclinical studies demonstrated that the combination of bevacizumab and pembrolizumab had a synergistic effect in both advanced tumor therapy and neoadjuvant setting and therefore provide a theoretical basis for translating this basic research into clinical applications.

**Supplementary Information:**

The online version contains supplementary material available at 10.1007/s00262-022-03318-x.

## Introduction

Lung cancer is the malignant tumor with the highest morbidity and mortality rates in the world [1]. Non-small cell lung cancer (NSCLC) accounts for approximately 85% of all lung cancer cases, with a 5-year overall survival rate of approximately 15%. Most patients diagnosed with NSCLC are in advanced stages and are not candidates for curative surgery [2]. The development of immune checkpoint inhibitors (ICIs) has ushered in a new era in the treatment of NSCLC, following that of chemotherapy and targeted therapy [3]. The clinical application of various ICIs has led to dramatic changes in the treatment for NSCLC patients. However, the fact that only a small subset of patients with specific tumor microenvironment can benefit from ICIs limits their application [4]. For instance, only tumors with pre-existing immunity (i.e., many tumor-infiltrating lymphocytes, dense CD8^+^ T cells, and high PD-L1 expression) respond well to ICIs. Other phenotypes, such as immune-excluded tumors (immune cells only present at the periphery) and ‘cold’ tumors (little or no immune-cell infiltration), respond poorly to single-dose ICIs [5, 6]. Unfortunately, the immune infiltration of most NSCLC is characterized by immune exclusion [7]. Therefore, a suitable combination therapy is needed to increase the infiltration of tumor antigen-specific T cells in malignant tumor tissues and then, combined with ICIs, to reverse tumor-induced immunosuppression and destroy tumor cells [8].

Among the many options for combination therapy strategies, targeting angiogenesis appears to be a promising direction. Angiogenesis contributes to tumorigenesis, tumor progression, and metastasis in numerous malignancies [9, 10]. Vascular epithelial growth factor (VEGF) is a main regulator of angiogenesis, which stimulates the proliferation, migration, and neovascularization of vascular epithelial cells by binding to VEGF receptors [11]. However, abnormal neovascularization (stiffness, distortion, dilatation, and structural abnormalities) and low pericyte coverage can result in an adverse microenvironment with nutrient disorder, hypoxic, acidic and interstitial pressure [12]. Notably, the VEGF pathway can also contributes to the formation of an inhibitory immune microenvironment [13]. For instance, aberrant expression of VEGF can prevent the trafficking of tumor-reactive T cells to the tumor site by inhibiting the expression of adhesion molecules, such as intercellular adhesion molecule 1 (ICAM-1) and vascular cell adhesion molecule 1 (VCAM-1) within endothelial cells [14]. Given this, bevacizumab, a monoclonal antibody against VEGF, was developed and first approved by the United States Food and Drug Administration (FDA) for the treatment of metastatic colorectal cancer in 2004 [15]. Since then, it has shown survival benefits in a variety of solid tumors including NSCLC [16, 17]. Therefore, we postulate that, in addition to the function of anti-angiogenic agents, the immunomodulatory properties of bevacizumab may also play a role in its clinical activity.

In fact, many clinical trials of anti-angiogenic drugs and ICIs combination therapy are underway, and some preclinical studies have also been conducted on various tumor models and have shown promise [18]. Kohei et al. [19] found that in a mouse model of hepatocellular carcinoma, anti-VEGFR2 antibody-mediated vascular normalization can promote the efficacy of ICIs by reprogramming the immune microenvironment. However, these preclinical models lack the human immune system and therefore unable to use clinical antibodies such as pembrolizumab. In this regard, the emergence of humanized immune system in mice has brought new hope for preclinical immunotherapy research. Human peripheral blood mononuclear cell (PBMC) and human hematopoietic stem cell (HSC) mouse models were established by transplanting PBMCs or cord blood-derived CD34^+^ HSCs into severe combined immunodeficiency mic [20]. In addition, tumor cell lines or patient-derived xenografts could also transplanted into mice. Humanized mouse models are essential for preclinical testing of immunotherapies, as they provide insight into the interactions between the human immune system and tumors [21]. Among the two humanized mouse models, we mainly focus on the role of T cells in tumor immunity, so we chose the humanized PBMC model, whose reconstructed immune system is dominated by T cells.

Notably, although ICIs monotherapy or combination therapy can significantly improve the prognosis of patients with advanced NSCLC, there is still a lack of stubborn clinical evidence on whether these treatment can be applied to neoadjuvant therapy in patients with early-stage lung cancer [22]. For example, the effect of neoadjuvant pembrolizumab plus bevacizumab in NSCLC and the possible side effects, such as wound healing complications (WHC) and surgical site bleeding, require further study. In this regard, mouse models are potential alternatives due to their relative short life and feasibility to observe long-term survival after neoadjuvant therapy. Traditionally, neoadjuvant mouse models usually select cell lines that metastasize spontaneously, such as 4T1.2 and E0771 cell lines, to mimic clinical recurrence and metastasis after surgical resection [23]. However, there is no human NSCLC cell line capable of spontaneous metastasis. This underlines the importance of constructing optimal animal model capable of conducting neoadjuvant/adjuvant immunotherapy experiments for NSCLC.

In this study, we hypothesized that combined bevacizumab and pembrolizumab therapy can inhibit tumor growth by inducing activated T cell infiltration, as well as produce systemic immunity to eliminate recurrence and metastasis. To this end, we established a human PBMC (Hu-PBMC) mouse model, as well as neoadjuvant mouse model, to conduct preclinical immunotherapy study. Our preclinical findings suggest that combination therapy has a synergistic antitumor effect in both advanced tumor and neoadjuvant setting, providing a theoretical basis for its first-line clinical application.

## Materials and methods

### Animals and cell lines

Female B-NDG (NOD-Prkdc scid IL2rg tm1/Bcgen) mice (5–6 weeks old) were obtained from Biocytogen (Beijing, China). The mice were given autoclaved water and food under specific pathogen-free conditions. Mice were humanely euthanized by CO_2_ inhalation if a solitary subcutaneous tumor exceeded 1500 mm^3^ in size. Animal experiments were performed at the Laboratory Animal Center of the Air Force Military Medical University (Xi'an, China) and followed the protocol approved by the Institutional Animal Care and Use Committee (Approval Number: IACUC-20200602).

The human NSCLC cell lines (H1299 and A549) were purchased from the Cell Bank of the Chinese Academy of Sciences (Shanghai, China). Cells were cultured in RPMI-1640 medium (Thermo Fisher Scientific, Waltham, MA, USA) supplemented with 10% fetal bovine serum (Gibco, Grand Island, NY, USA) and 1% penicillin/streptomycin (Thermo Fisher Scientific) and incubated at 37 °C with 5% CO_2_. The identity of the cell lines was confirmed with STR profiling (Promega) on an ad hoc basis. When 70% confluent, the cells were suspended in serum-free medium with Matrigel (BD Biosciences, San Jose, CA, USA). 5 × 10^6^ cancer cells were injected subcutaneously into the humanized mice for in vivo studies.

### Antibodies

Humanized mice were treated with bevacizumab (anti-VEGF antibody) (Roche, Basel, Switzerland) and pembrolizumab (anti-PD-1 antibody) (Merck, Whitehouse Station, NJ, USA). For flow cytometry, we carefully incubated single-cell suspensions with anti-human CD45-fluorescein isothiocyanate (clone HI30), and anti-human CD3-phycoerythrin (clone UCHT1) antibodies (BD Biosciences). Immunohistochemistry was performed using anti-human CD45 (ab40763; 1:250 dilution), anti-human CD4 (ab133616; 1:500 dilution), and anti-human CD8 (ab108343; 1:400 dilution) antibodies (Abcam, Cambridge, MA, USA). Anti-mouse CD31 (77,699; 1:100 dilution) antibodies were purchased from Cell Signalling Technology (Danvers, MA, USA). Immunofluorescence analysis was performed using anti-mouse CD31 (3528; 1:2000 dilution) (Cell Signalling Technology), anti-human CD45 (ab40763; 1:100 dilution) (Abcam), anti-human CD4 (ab196372; 1:50 dilution) (Abcam), anti-human CD8 (ab237709; 1:100 dilution) (Abcam), anti-mouse ICAM-1 (ab222736; 1:50 dilution) (Abcam), anti-mouse VCAM-1 (ab134047; 1:250 dilution) (Abcam), anti-human granzyme B (ab208586; 1:250 dilution) (Abcam), anti-human PD-1 (ab234444; 1:50 dilution) (Abcam), anti-human TNF-alpha (ab215188; 1:100 dilution) (Abcam) and anti-mouse alpha-smooth muscle actin (*α*-SMA) (36,110; 1:50 dilution) (Cell Signalling Technology) antibodies. The human CD8-α monoclonal antibody (clone OKT-T8) (BioXcell, West Lebanon, NH, USA). Human CD8^+^ T cells were depleted by injecting (ip) B-NDG mice with 200 mg anti-CD8 depletion mAb (clone OKT-T8, BioXcell, West Lebanon, NH, USA) 2 days before treatment and then followed by weekly intraperitoneal injections of anti-CD8 mAb for 2 weeks.

### Animal experimental protocol

Humanized mice were developed as described previously [19]. Briefly, two fresh peripheral blood samples were collected from the Blood Transfusion Department of Xijing Hospital (Xi'an, China). The protocol was strictly obeyed under the guide of Medical Ethics Committee (Approval Number: KY20193035). Whole PBMCs were isolated using Lymphoprep (Axis-Shield, Dundee, UK) and with the manufacturer’s instructions. Hu-PBMC mice were developed by intravenously injecting 1 × 10^7^ human PBMCs into 6-weeks-old female B-NDG mice. The engraftment levels of human CD45^+^ CD3^+^ cells were determined 3 weeks after PBMC transplantation with flow cytometric quantification. Mice with ≥ 25% were considered engrafted and humanized. Humanized mice derived from different PBMC donors with diverse levels of human CD45^+^ CD3^+^ cells were randomly assigned to each treatment group in each experiment.

Tumor size, which was determined every 3–4 days using an electric calliper, was calculated as follows: volume (mm^3^) = (length × width^2^)/2. When the tumor size reached 1500 mm^3^, the mice were then sacrificed. Hu-PBMC cell line-derived xenograft (CDX) mice were treated as follows, with doses determined based on our preliminary experiments and previous studies. In the control group, mice received immunoglobulin G 21 days after PBMC transplantation. In the bevacizumab monotherapy group, bevacizumab (1 mg/kg, intraperitoneally, once every 3 days) was administered 21 days after PBMC transplantation. In the pembrolizumab monotherapy group, pembrolizumab (10 mg/kg, intraperitoneally, once every 3 days) was administered 24 days after PBMC transplantation. In the combination group, bevacizumab was administered 21 days after PBMC transplantation, followed by pembrolizumab on day 24.

### Flow cytometry

Two weeks after PBMC transplantation, the peripheral blood collected from the tail vein of mice was detect the content of the humanized level. At the completion of the study, mice were euthanized by CO_2_ inhalation. The spleen and bone marrow were collected immediately after euthanasia. Cell acquisition was performed with a FC500 flow cytometer (Beckman Coulter, Miami, FL, USA). Lastly, data were analyzed using FlowJo (version 10.7) (TreeStar, San Carlos, CA, USA).

### Immunohistochemistry and immunofluorescence

Tumors, spleens, and bone marrows harvested from humanized mice were fixed in 10% formalin and then embedded with paraffin. Tumors were then cut into 5-mm sections and subjected to standard hematoxylin and eosin staining or immunohistochemistry. Histology slides were scanned with the Aperio imaging system (Leica Biosystems, Buffalo Grove, USA) and analyzed using ImageScope (Leica Biosystems).

For immunofluorescence analysis, sections were deparaffinized, rehydrated, and boiled in a microwave for 15 min in 10 mM citrate buffer for antigen retrieval. Staining was performed using the standard procedure and aforementioned antibodies. The slides were imaged using an SP8 confocal microscope (Leica Microsystems, Wetzlar, Germany). Tumor vessel density was determined based on CD31^+^ luminal structures. The count of immune cells around blood vessels is to select multiple CD31^+^ vascular regions within tumor and calculate the number of immune cells in a certain area around the vessel.

### Statistical analysis

All statistical analyses were performed with GraphPad Prism (version 7.0) (GraphPad Software, Inc., San Diego, CA, USA). Data are expressed as means ± standard deviation. Differences between multiple groups were tested with analysis of variance and the differences between two groups were analysis with LSD *t*-test. *P* < 0.05 was considered significant.

## Results

### Combined bevacizumab and pembrolizumab therapy induces synergistic antitumor effects in NSCLC irrespective of PD-L1 expression

**Fig. 1:**
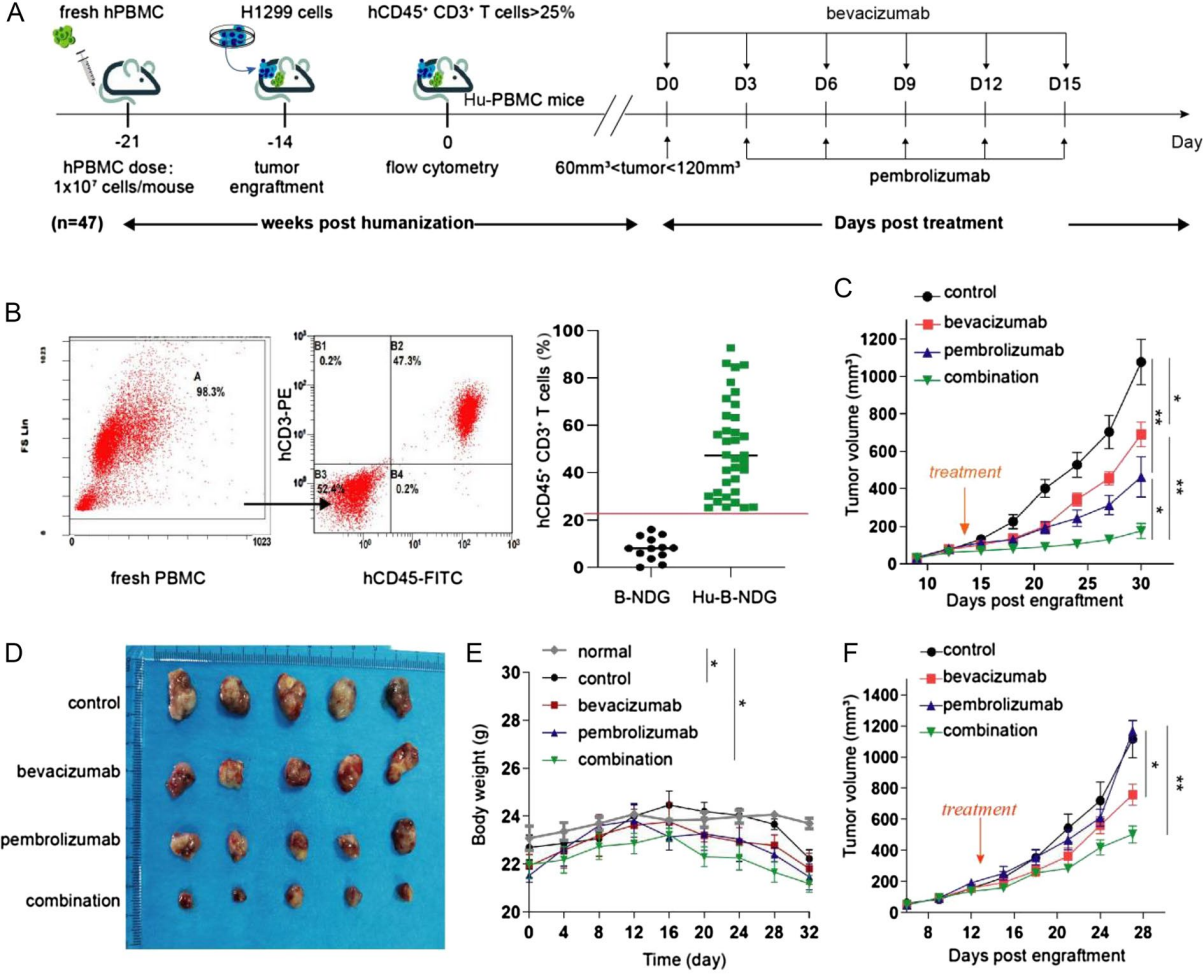
Efficacy of combined bevacizumab and pembrolizumab therapy in humanized mouse models of non-small cell lung cancer. **a** Schematic diagram of the construction and treatment of humanized mice. Adult B-NDG mice were injected with peripheral blood mononuclear cells intravenously, followed by subcutaneous transplantation of tumor cell lines. When the tumor reaches the therapeutic volume, the mice were randomly assigned to each treatment group. **b** The level of human CD45^+^CD3^+^ T cells in the blood of mice was monitored with flow cytometry, and the mice with a reconstruction level higher than 25% were selected for follow-up experiments. **c** Tumor growth curves of humanized mice bearing H1299 cells in the control, bevacizumab monotherapy, pembrolizumab monotherapy, and combination therapy groups. Arrow indicates start of treatment. Data are presented as the mean ± standard deviation (*n* = 5). *P < 0.05, and **P < 0.01. **d** At the end of treatment, the tumors were collected and photographed. **e** The body weight of the mice in each treatment group and normal B-NDG with no PBMCs transplantation was monitored every 4 days from the beginning of peripheral blood mononuclear cell transplantation to the end of treatment (*n* = 5). *P < 0.05. **f** Tumor growth curves of humanized mice bearing A549 cells in each of the four treatment groups after transplanted with PBMCs. Data are presented as the mean ± standard deviation (*n* = 5). *P < 0.05, and **P < 0.01

To investigate the therapeutic effect of the combination of bevacizumab and pembrolizumab, we transplanted human PBMCs and H1299 cells into 47 B-NDG mice to construct humanized mouse model of NSCLC (Fig. 1a). Three weeks later, we monitored the level of human CD45^+^ CD3^+^ T cells in the peripheral blood of mice, which are the main populations of immune cells in Hu-PBMCs mice (> 90%, data not shown). Next, 35 successfully reconstructed mice (CD45^+^ CD3^+^ T cell > 25%) were divided into four groups: control, bevacizumab monotherapy, pembrolizumab monotherapy, and combined bevacizumab and pembrolizumab therapy (Fig. 1b). When the volume of tumor reached 60–120 mm^3^, the treatment commenced and the scheme is illustrated. Over the course of treatment, we found that when administered alone, both bevacizumab and pembrolizumab reduced tumor volumes compared with those of untreated mice. Notably, the combination of bevacizumab and pembrolizumab reduced tumor volumes to a greater extent than either of the two monotherapies (Fig. 1c). At the end of treatment, we collected and compared the tumors in each group (Fig. 1d). To evaluate the safety of combination therapy, the body weight of the mice in each group was dynamically monitored. All humanized mice have been reported to experience weight loss due to graft-versus-host disease about 3 weeks after PBMCs transplantation. However, mice treated with the combination option did not experience additional weight loss compared to control group (Fig. 1e).

Furthermore, to study whether the effect of the combination therapy was dependent on the level of PD-L1 expression, we also conducted a combination therapy with A549 cell lines, which have low levels of PD-L1 expression [24]. The results showed that pembrolizumab monotherapy was ineffective, while bevacizumab monotherapy and combination therapy could inhibit tumor growth. This suggested that the combination therapy was also effective in treating tumors with low PD-L1 expression (Fig. 1f). Taken together, these results indicate that combination therapy can enhance antitumor activity irrespective of PD-L1 expression and without additional toxicity.

### Combined bevacizumab and pembrolizumab therapy reprograms the tumor immune microenvironment

**Fig. 2:**
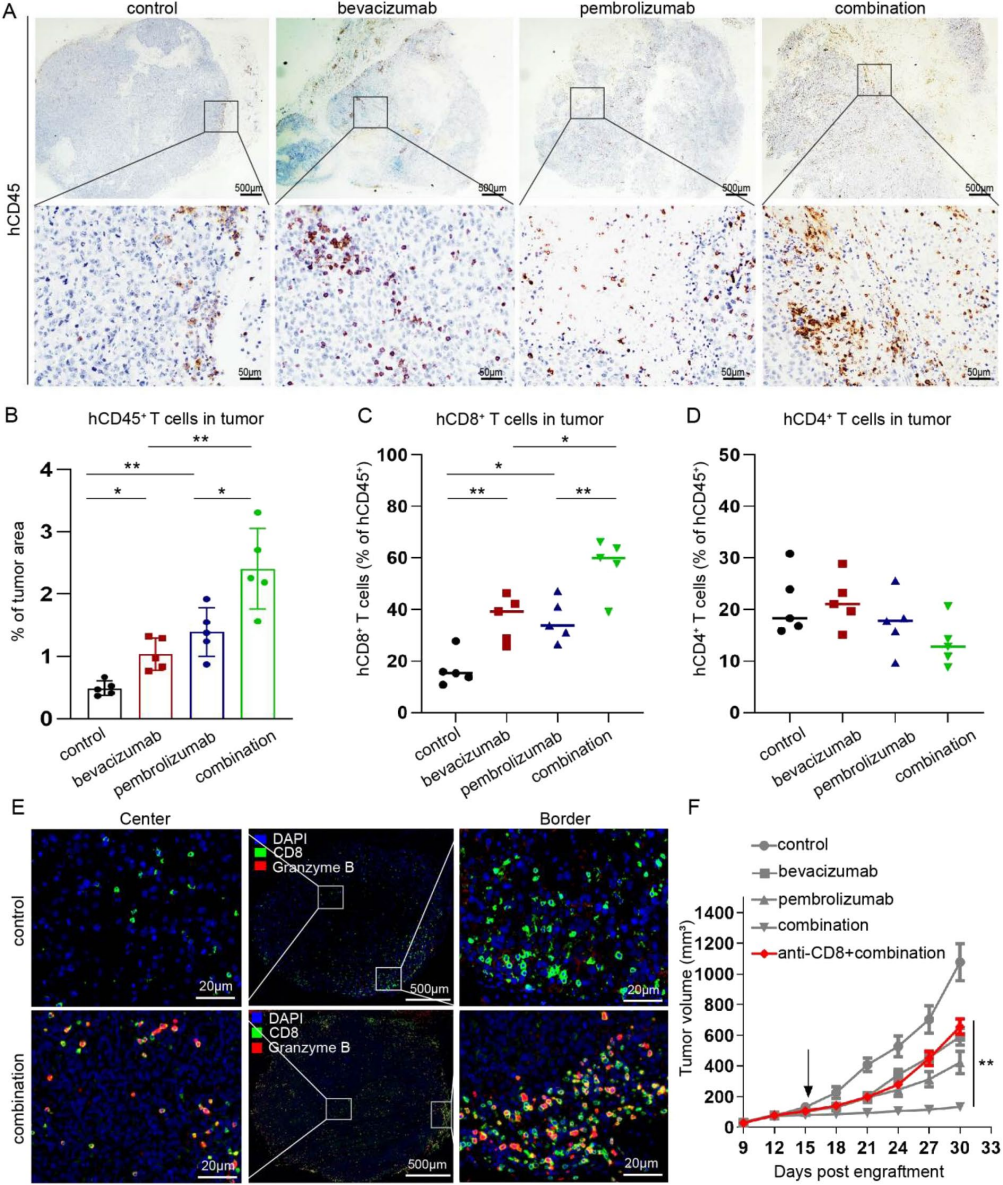
Impact of combined bevacizumab and pembrolizumab therapy on the tumor immune microenvironment. **a** Immunohistochemical staining of CD45^+^ cells in tumors treated as indicated. Representative IHC images are obtained at 2 × and 20 × magnification, respectively. **b** Quantification of CD45^+^ cells in H1299 tumors treated with IgG, bevacizumab, pembrolizumab, and combination therapy (*n* = 5). Each dot represents the mean of three random field in one sample. *P < 0.05, and **P < 0.01. **c**–**d** Flow cytometry analysis of the infiltration of CD4^+^ and CD8^+^T cells in tumors treated with IgG, bevacizumab, pembrolizumab, and combination therapy (*n* = 5). *P < 0.05, and **P < 0.01. **e** Immunofluorescence analysis of the distribution of CD8^+^ T cells (green) and granzyme B^+^ (red) in the center and at the periphery of tumor tissue in the control and combination therapy groups. Scale bars, 500 μm (low-magnification images); 20 μm (high-magnification images). **f** Tumor growth curves of humanized mice treated with anti-CD8-depleting monoclonal antibody plus combination therapy (red), as well as above mentioned tumor growth curves of four groups (grey). Arrow indicates start of treatment. Data are presented as the mean ± standard deviation (n = 5). **P < 0.01

To analyze the underlying mechanisms of tumor growth inhibition induced by combined PD-1 and VEGF blockade in humanized mice bearing H1299 cells, we first examined the effect of treatment on lymphocyte infiltration using immunohistochemistry. Histochemical results showed that both of the two monotherapy groups increased the number of infiltrating immune cells in the tumor to varying degrees when compared with control group. Further, when compared with monotherapy groups, the combination therapy transformed the tumor from “immune excluded” to “hot” tumor, as demonstrated by increased CD45^+^ immune cells in both tumor border and center (Figs. 2a, b and S1a). To further accurately detect the changes of immune cells in the tumor, the number of CD8^+^ and CD4^+^ T cells in the tumor were analyzed by flow cytometry. Results showed that the number of CD8^+^ tumor-infiltrating lymphocytes in the two monotherapy groups were higher than that in the control group, the combination therapy group exhibit highest CD8^+^ T cell infiltrating when compared with two monotherapy groups (Fig. 2c). However, the numbers of CD4^+^ T cells in four groups were not statistically significant (Fig. 2d). Next, to characterize the phenotype of altered CD8^+^ T cells in tumor, we tested the activity of intratumoral CD8^+^ T cells between control and combination groups. Results showed that CD8^+^ T cells exhibited significantly higher amounts of granzyme B proteins in the combination group, which represent the activation of CD8^+^ T cells (Fig. 2f). So with the increase of CD8^+^PD-1^+^ T cells and CD8^+^TNF-alpha^+^ T cells in the combination group than in the control group (Figure S1c-d). These results suggested that combination therapy can restore the activity of CD8^+^ immune cells and therefore destroy tumor cells. To study whether CD8^+^ T cells mediate the effect of combination therapy, we pre-treated humanized mice with anti-CD8 antibody to eliminate CD8^+^ T cells in vivo and then administered the combined therapy. The results showed the depletion of CD8^+^ T cells on combination group abrogated the effect of pembrolizumab in combined therapy, this suggested that the promoting effect of bevacizumab on pembrolizumab is mainly mediated by CD8^+^ T cells (Figs. 2e and S1b). Taken together, these results highlight the important role of activated cytotoxic CD8^+^ T cells within tumor for combination therapy.

### Reprogramming of the tumor immune microenvironment is mediated by vascular normalization

**Fig. 3:**
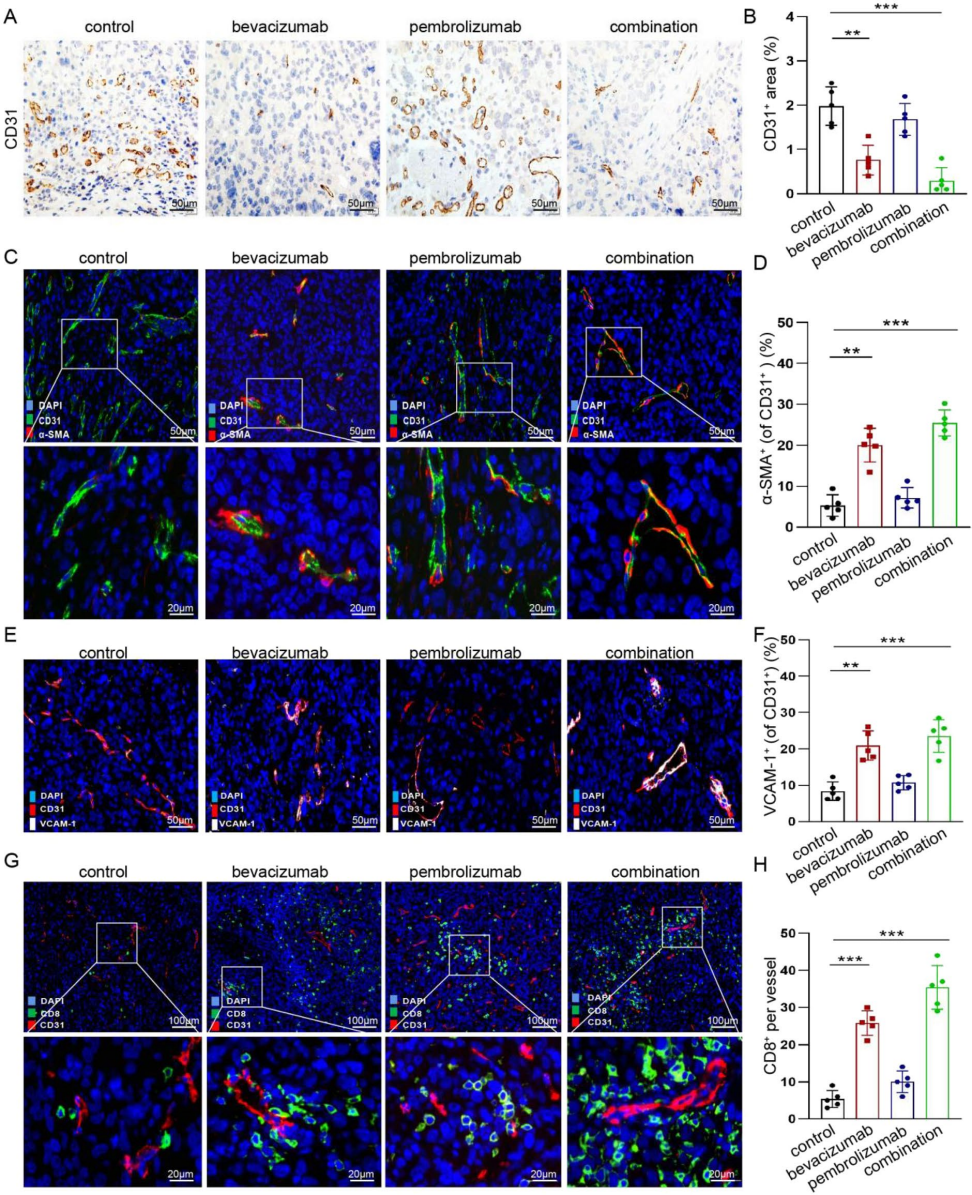
The effect of vascular normalization on tumor immune microenvironment. **a**, **b** Immunohistochemical analysis and quantification of CD31^+^ areas in tumors of four groups. Representative images are obtained at × 20 magnification. The IHC results of the four groups were quantified and compared. Each dot represents the mean of three random field in one sample (*n* = 5). **P < 0.01, and ***P < 0.001. **c**, **d** Representative images of CD31^+^ (green) and α-smooth muscle actin (*α*-SMA)^+^ (red) immunostaining of tumors in four groups. Scale bars, 50 μm (low-magnification images); 20 μm (high-magnification images). The histochemical results of the four groups were quantified and compared. Each dot represents the mean of three random field in one sample (*n* = 5). **P < 0.01, and ***P < 0.001. **e**, **f** Immunofluorescence analysis and quantification of VCAM-1^+^ (white) and CD31^+^ (red) areas in tumors of four groups. Representative images are obtained at × 20 magnification. The immunofluorescence staining results of the four groups were quantified and compared. Each dot represents the mean of three random field in one sample (*n* = 5). **P < 0.01, and ***P < 0.001. **g**, **h** Immunofluorescence analysis and quantification of CD31^+^ (red) and CD8^+^ (green) in tumors of four groups. Representative images are obtained at × 10 magnification. The immunofluorescence staining results of the four groups were quantified and compared. Each dot represents the mean of three random field in one sample (*n* = 5). ***P < 0.001

To gain insight into the mechanism by which combination therapy induces "immune hot" tumor microenvironment, we further analyzed the changes of blood vessels and its relationship with immune cells. Firstly, tissue sections from each group were stained by immunohistochemistry and immunofluorescence for vascular marker CD31. Bevacizumab alone or in combination with pembrolizumab resulted in a significantly smaller vascular area than either the pembrolizumab or control groups (Fig. 3a, b). To better define the effect of these therapies on tumor vasculature, we performed immunofluorescence analysis and measured the vascular area in the center and periphery of tumor. At the periphery, bevacizumab monotherapy reduced the CD31^+^ vascular area, whereas the combination therapy resulted in a significantly smaller vascular area than either the monotherapy or control. In the center, the CD31^+^ vascular area in each group showed the same trend as the periphery (Fig. S2a and b). Next, to assess vascular maturation, pericytes were visualized with α-SMA immunostaining. Consistent with the microvessel density, bevacizumab alone or in combination with pembrolizumab increased the pericyte coverage of the surviving tumor vessels (Fig. 3c, d). Furthermore, VCAM-1 expression was detected in the endothelial cells of each group to determine whether endothelial cell adhesion function had recovered. The proportion of VCAM-1^+^ CD31^+^ cells in the bevacizumab monotherapy and combination therapy groups was significantly higher than that in the pembrolizumab monotherapy or control group (Fig. 3e, f). Similarly, the ICAM-1 expression and its co-expression with CD31 was also tested, and the result is similar to VCAM-1 (Fig. S2c and d). All these results suggest that the bevacizumab monotherapy therapy and the combination therapy can promote the normalization of tumor vasculature.

A major reason for the formation of immune-excluded tumors is that the tumor vasculature inhibits the extravasation of lymphocyte. Therefore, we hypothesized that tumor vasculature normalization may be partially responsible for the different infiltration levels of CD8^+^ T cells across the groups. The results of immunofluorescence staining showed that bevacizumab alone or in combination with pembrolizumab specifically increased the absolute number of perivascular CD8^+^ T cells (Fig. 3g). This suggests that vascular normalization does increase the infiltration of CD8^+^ cells around blood vessels. Quantitative morphometric analysis of tumor sections also confirmed these results (Fig. 3h). Finally, we also investigated the effect of vascular normalization in the compactness and oxygen supply of tumor. Specifically, H&E staining results showed that the unpacked tumor (necrosis area) was larger in three treatment groups than in the control group (Fig. S2e). In addition, we measured hypoxia in tumors with immunofluorescence staining of hypoxia-inducible factor-1 α (HIF-1 α). Results showed that about 50% area of tumors in control group showed anoxic markers in positive. While the combination therapy only account for about 20% (Fig. S2f). Taken together, these results suggest that vascular normalization can reprogram the tumor microenvironment, in particular, to promote the recruitment and infiltration of CD8^+^ T cells.

### Construction and identification of neoadjuvant immunotherapy mouse models

**Fig. 4:**
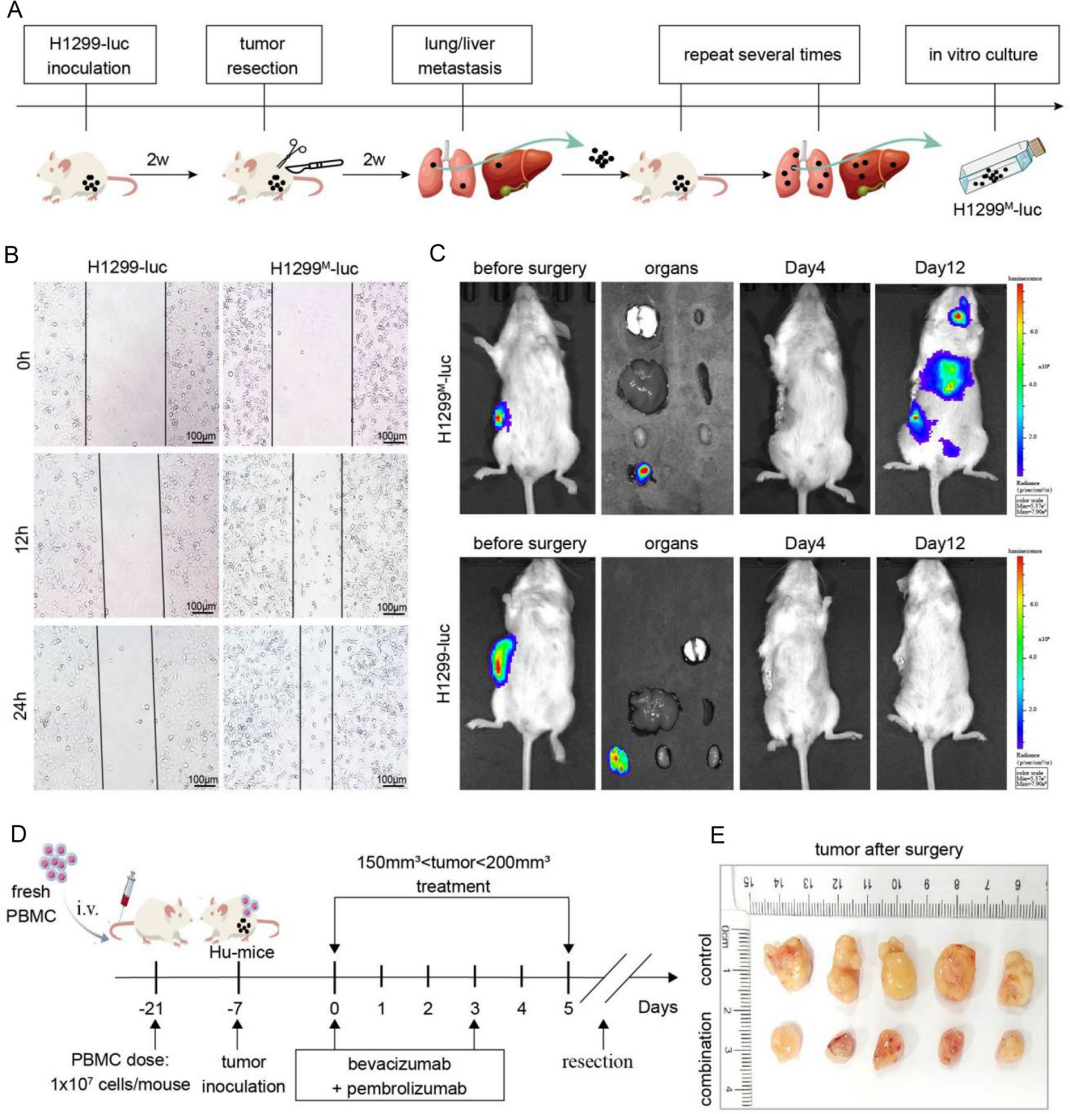
Construction and identification of neoadjuvant immunotherapy mouse models. **a** The H1299-Luc cell line with high metastatic properties was obtained by serial screening between mouse subcutaneous and liver and lung metastases, and specific screening process is shown in Figure. **b** The scratch assay was performed to further determine the high metastatic properties of the obtained H1299^M^-luc. Specifically, the width of scratched areas were measured after 0, 12 and 24 h of scratch, to analyze the migration ability of H1299^M^-luc cells. **c** An in vivo imaging system was used to observe the metastatic ability of the H1299^M^-luc cell line and its paternal line after complete subcutaneous tumor resection, a process that simulates recurrence and metastasis after clinical surgical resection. **d** When tumors reached a certain volume, mice were treated with bevacizumab combined with pembrolizumab in neoadjuvant setting for 5 days, followed by absolute subcutaneous tumor resection. The specific experimental process is shown in Figure. **e** The surgically removed subcutaneous tumor was collected and photographed

Since combination therapy can control local tumors by reprogramming the immune microenvironment, whether it can induce a systemic immune response to prevent postoperative recurrence and metastasis deserves attention. To solve this problem, appropriate mouse models of neoadjuvant immunotherapy are necessary. H1299-Luc cells were transplanted subcutaneously into mice, then excised half of the tumor when it grew to a certain volume, executed the mice after a period of time and collected the metastases of lung and liver, then transplanted the metastases into the subcutis of another mouse. Repeat this process several times and we can obtain a highly metastatic H1299-Luc cell line after in vitro culture of metastases of lung and liver (named H1299^M^-Luc) (Fig. 4a). In order to detect whether H1299^M^-Luc cells have high metastasis characteristics at the cellular level, we conduct Wound-Healing Assay with two types of cells and the results showed that H1299^M^-Luc cells had better migration ability than H1299-Luc cells (Fig. 4b). Further in vivo experiments showed that mice loaded with H1299^M^-Luc cells developed metastasis approximately 1 week after complete subcutaneous surgery (no visible residual tumor), while mice loaded with the normal H1299-Luc cells did not develop recurrence and metastasis after surgery (Fig. 4c). Next, we transplant this highly metastatic cell line into humanized mice to perform neoadjuvant immunotherapy. As shown in Fig. 4d, before the subcutaneous tumor was completely resection, the mice were treated with neoadjuvant combination therapy twice for 5 days, and the recurrence and metastasis of the control group and the combined treatment group were observed after surgery. In addition, we compared the difference in tumor volume at surgical resection between the control and combination groups (Fig. 4e). In conclusion, the mouse model constructed by us can be used for the study of neoadjuvant immunotherapy.

### Neoadjuvant combination therapy can prevents postoperative recurrence and metastasis

**Fig. 5:**
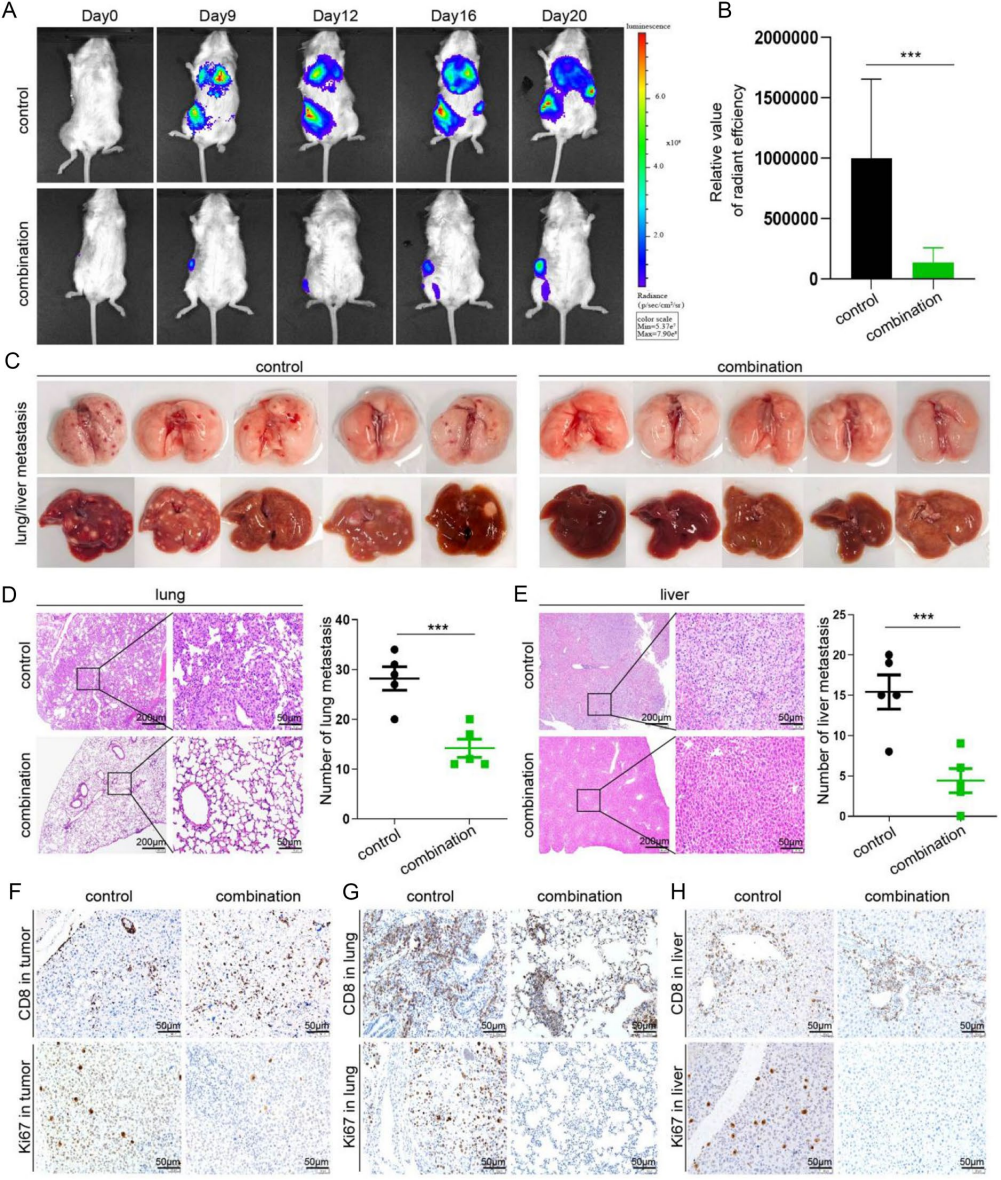
The effect of neoadjuvant combined therapy in preventing recurrence and metastasis. **a**, **b** The tumor recurrence and metastasis of the mice in the control group and the neoadjuvant combination treatment group were observed at 0, 9, 12, 16 and 20 days after the operation using the in vivo imaging system. The relative value of radiant efficiency between control group and neoadjuvant group was recorded and compared (*n* = 5). ***P < 0.001. **c** After the experiment, the mice were sacrificed and the lungs and livers of the two groups were collected and photographed. **d**, **e** H&E staining was performed on the livers and lungs of all mice in the two treatment groups, a representative area was selected to take pictures and record the number of metastases in each mice. Each dot represents the mean of three random field in one sample (*n* = 5). ***P < 0.001. **f**–**h** Immunohistochemical staining to analyze the number of CD8^+^ T cells and the expression of Ki67 in subcutaneous tumors, lung metastases and liver metastases in mice

Previous preclinical studies usually observed tumor recurrence and metastasis by sacrificing mice after therapy, but we used IVIS to dynamically observe tumor changes within mice after neoadjuvant combination therapy. As shown in Fig. 5a, neoadjuvant combination therapy clearly prevent metastasis and delayed the recurrence of tumor when compared with control group. Further quantitative analysis of IVIS also confirmed the effect of neoadjuvant combination therapy (Fig. 5b). Next, we sacrificed the mice and collected major organs such as lung and liver in each groups. Liver and lung were photographed to evaluate tumor metastasis on a macro scale; the number of metastases in the neoadjuvant combination therapy group was significantly lower than that in the control group (Fig. 5c). The finding was subsequently confirmed by histopathological evaluation of H&E stained section (Fig. 5d, e). Finally, given the central role of CD8^+^ T cells in antitumor immunity, we compared the infiltration of CD8^+^ T cells in subcutaneous tumors, lung metastases, and liver metastases in the control and neoadjuvant combination therapy groups. Histopathological results showed that neoadjuvant immunotherapy significantly increased the infiltration of CD8^+^ T cells in subcutaneous tumors, which was the same as the above-mentioned results. However, neoadjuvant combination therapy had no effect on the infiltration of CD8^+^ T cells in both liver and lung metastases. Notably, neoadjuvant therapy inhibited the proliferation of tumor cells in subcutaneous tumor as well as in metastatic foci, as demonstrated by significantly reduced Ki67 staining (Fig. 5f–h and S3). This suggests that neoadjuvant immunotherapy can improve the immune microenvironment of primary tumors but not metastases. Overall, our study demonstrated that neoadjuvant therapy can effectively slow postoperative recurrence and metastasis.

## Discussion

Our preclinical study investigated the synergistic effect and underlying mechanism of the bevacizumab and pembrolizumab in both advanced tumor therapy and neoadjuvant setting. We found that the combination of the two therapies could control local tumor growth by increasing CD8^+^ T cell infiltration. In addition, based on the neoadjuvant immunotherapy mouse model, we also tested the effect of this combination as a neoadjuvant therapy to prevent recurrence and metastasis and therefore provided a crucial theoretical basis for subsequent clinical trials.

Immunotherapy has entered the era of combination therapy, and the combination of ICIs and anti-angiogenic drugs seems to be a promising strategy. In fact, several preclinical trials have reported that targeting VEGF or VEGFR2 can improve the efficacy of immunotherapy. For instance, Allen et al. combined anti-VEGFR2 antibody and anti-PD-L1 antibody in several tumor mouse models and found the antiangiogenic therapy can sensitize tumors to ICIs therapy specifically by generating intratumoral high endothelial venules (HEVs) that facilitate enhanced T cells infiltration, activity, and tumor cell destruction [25]. Nevertheless, these studies did not further reveal the distribution of CD3^+^ and CD8^+^ T cells within tumor. Our study founded that bevacizumab can increase the level of activated CD8^+^ T cells in both center and periphery of the tumor by normalizing vessels, thereby transforming NSCLC into a "hot tumor" that is more susceptible to ICIs. Notably, another problem with these studies is that the tumors, antibodies, and immune system used came from mice rather than humans and thus have to be prudent in directly translating these results to patients. Therefore, it is of great significance to study the combined treatment effect of the most commonly used targeted angiogenesis drug (bevacizumab) and immune checkpoint inhibitor (pembrolizumab) in clinical practice.

Clinically, neoadjuvant pembrolizumab in melanoma has yielded promising results, with high rates of pathologic complete response (pCR) and improved relapse-free survival rates [26]. As for NSCLC, there are few clinical trial testing whether neoadjuvant immunotherapy combined with bevacizumab can achieve high rates of major pathologic response (MPR) or prolong overall survival (OS). To solve these problem, we innovatively constructed a mouse model of neoadjuvant immunotherapy based on humanized mice. The mouse model was observed to experience recurrence and metastasis approximately 1 week after complete subcutaneous resection of the tumor, which perfectly mimic the clinical process after surgery. In addition, the end-point event MPR in most previous clinical trials did not intuitively predict postoperative recurrence and metastasis in neoadjuvant patients [27]. The preclinical model we constructed can dynamically observe tumor recurrence and metastasis through the IVIS. The results showed that the neoadjuvant combination therapy significantly prevented postoperative recurrence and metastasis compared with the control group. This exciting results demonstrates the feasibility of bevacizumab combined with pembrolizumab as a neoadjuvant therapy and provides a theoretical basis for subsequent clinical trials. However, there are still some clinical issues need to be addressed during the clinical translation of our study. For example, previous studies have reported that application of bevacizumab in perioperative period may cause delayed wound healing and bleeding [28]. For this reason, most physicians recommend an interval of ≥ 4 weeks before or after surgery, as determined by the half-life of bevacizumab (approximately 20 days) [29, 30]. Clinically, current trials of neoadjuvant bevacizumab combined with immunotherapy (NCT04973293) or with chemotherapy (NCT00025389) have chosen to perform surgery at 4–6 weeks after completion of perioperative therapy to avoid these complications.

The application of the neoadjuvant immunotherapy mouse model is not limited to exploring the effect of combination therapy; it can also help to (1) exploring biomarkers that can predict the effect of neoadjuvant combination therapy in NSCLC patients [31], (2) optimizing the optimal number of therapy cycles prior to surgery, (3) identifying appropriate blood markers that can help determine the optimal timing of surgery, such as CD8^+^ PD-1^+^ T cells etc., (4) defining the role of adjuvant immunotherapy and detailed therapy schedule, (5) using liquid biopsy to early predict metastatic and recurrence [32]. In addition, the immune system of a humanized mouse model constructed using PBMCs is mainly composed of human T cells. Other key immune subpopulations, such as B cells and natural killer cells are lacking [33]. In the follow-up experiments, we will use the humanized mouse model constructed by HSCs to further study the role of other immune subsets in combination therapy.

In conclusion, there is an urgent need for novel combination therapies in both advanced tumor therapy and neoadjuvant setting. In this regard, our results can be of high translational value since we identified the underlying mechanisms of combining immunotherapy with anti-angiogenic treatment. Our study provides powerful preclinical evidence for the effectiveness of bevacizumab plus pembrolizumab as first-line therapy in advanced NSCLC or neoadjuvant therapy in early NSCLC.

## Supplementary Information

Below is the link to the electronic supplementary material.

Supplementary file1 (DOCX 536 KB)

## Data Availability

The data that support the findings of this study are available from the corresponding author upon reasonable request.
