# Correction to: Combined pembrolizumab and bevacizumab therapy effectively inhibits non-small-cell lung cancer growth and prevents postoperative recurrence and metastasis in humanized mouse model

**DOI:** 10.1007/s00262-022-03342-x

**Published:** 2022-12-26

**Authors:** Tianyun Qiao, Jinbo Zhao, Xiangbing Xin, Yanlu Xiong, Wenwen Guo, Fancheng Meng, Hui Li, Yangbo Feng, Hui Xu, Changhong Shi, Yong Han

**Affiliations:** 1 Department of Thoracic Surgery, Air Force Medical Center, Beijing, 100142 China; 2 Department of Thoracic Surgery, Tangdu Hospital, Fourth Military Medical University, Xi’an, 710032 China; 3 Laboratory Animal Center, Fourth Military Medical University, Xi’an, 710032 China; 4 School of Basic Medicine, Fourth Military Medical University, Xi’an, 710032 China

**Correction to: Cancer Immunology, Immunotherapy** 10.1007/s00262-022-03318-x

In the online published article, the affiliation details for Authors Tianyun Qiao, Yangbo Feng and Yong Han were given as:

Department of Thoracic Surgery, Air Force Specialty Medical Center, Fourth Military Medical University, Xi’an 710032, China

but should have been

Department of Thoracic Surgery, Air Force Medical Center, Beijing 100142, China

