# Supplementary material for: Combined pembrolizumab and bevacizumab therapy effectively inhibits non-small-cell lung cancer growth and prevents postoperative recurrence and metastasis in humanized mouse model
Source: Cancer Immunol Immunother. 2022 Nov 10;72(5):1169–81. doi: 10.1007/s00262-022-03318-x (PMC10110651; doi:10.1007/s00262-022-03318-x)
Supplement: Supplementary file 1 — Supplementary file1 (DOCX 536 KB) [file 262_2022_3318_MOESM1_ESM.docx]

**
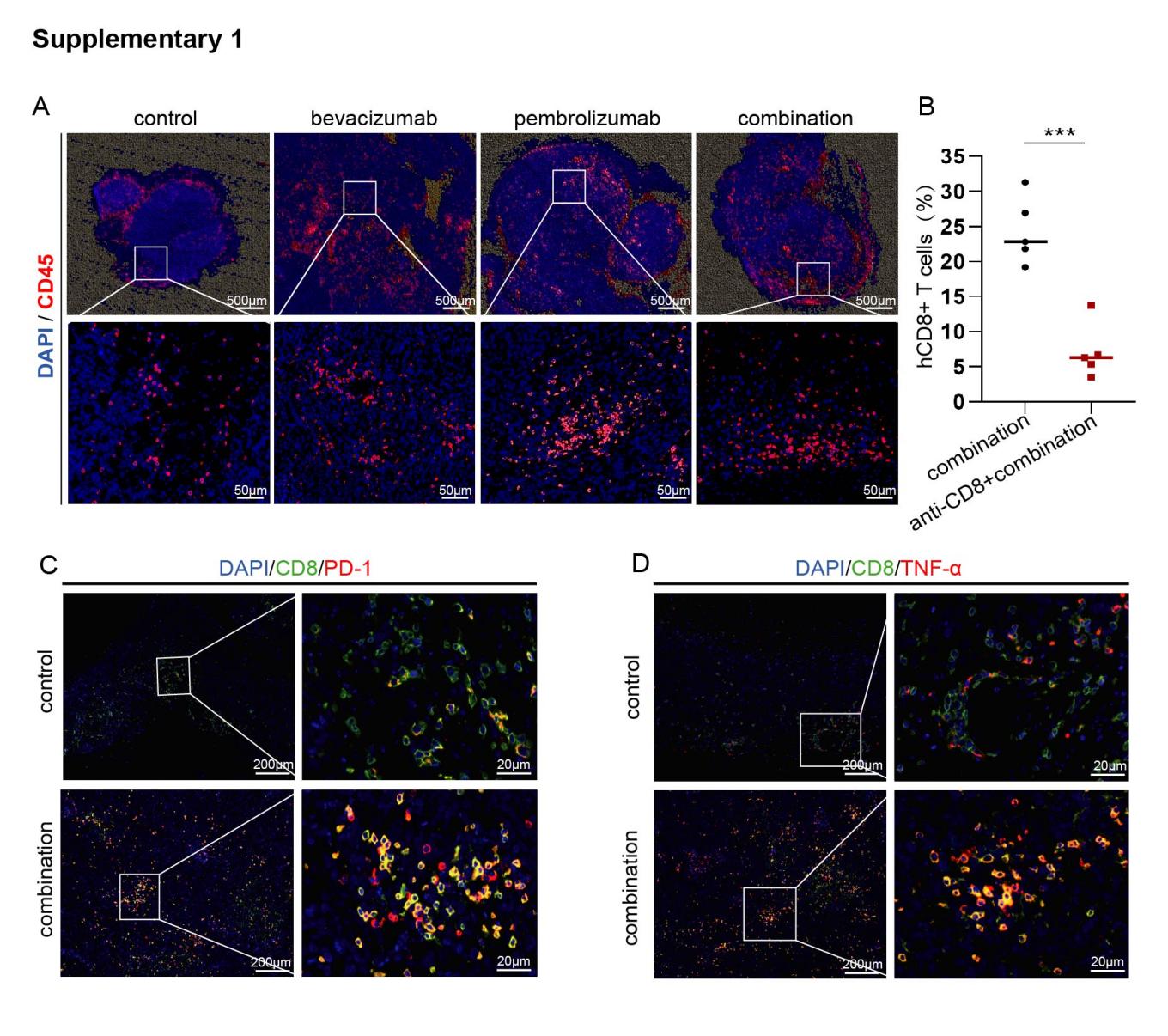
**

**Fig. S1. The effect of combined therapy in tumor immune microenvironment.** (**a**) Immunofluorescence staining of CD45^+^ cells in tumors treated as indicated. Representative images are obtained at ×2 and ×20 magnification respectively. The scale bar denotes 500μm and 50μm. (**b**) Using flow cytometry to determine the efficacy of adding CD8 in vivo deleting antibody to remove CD8^+^ T cells in the combination therapy group. (**c**) Immunofluorescence analysis of the distribution of CD8^+^ T cells (green) and PD-1^+^ (red) in the centre and at the periphery of tumor tissue in the control and combination therapy groups. Scale bars, 200 μm (low-magnification images); 20 μm (high-magnification images). (**d**) Immunofluorescence analysis of the distribution of CD8^+^ T cells (green) and TNF-α^+^ (red) in the centre and at the periphery of tumor tissue in the control and combination therapy groups. Scale bars, 200 μm (low-magnification images); 20 μm (high-magnification images).

**
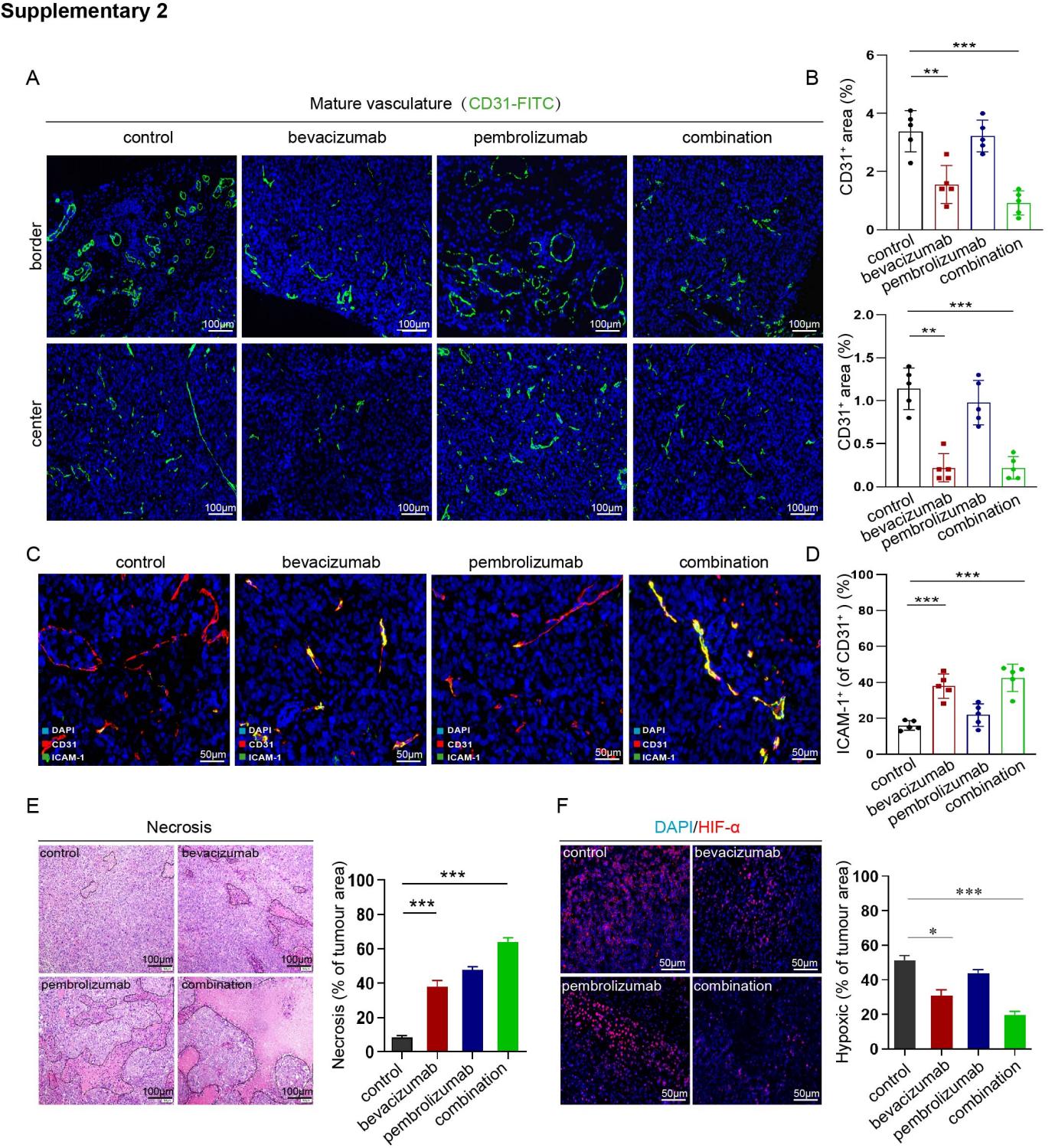
**

**Fig. S2. Bevacizumab monotherapy or combination therapy induces vascular normalization and improves tumor microenvironment.** (**a-b**) Immunofluorescence analysis and quantification of CD31^+^ areas in tumors border and center of four groups. Representative images are obtained at ×10 magnification. The scale bar denotes 100 μm. The results of the four groups were quantified and compared. ***P* < 0.01, and ****P* < 0.001. (**c-d**) Immunofluorescence analysis and quantification of ICAM-1^+^ (green) and CD31^+^ (red) areas in tumors of four groups. Representative images are obtained at ×20 magnification. The immunofluorescence staining results of the four groups were quantified and compared. ****P* < 0.001. (**e**) Effect of bevacizumab, pembrolizumab, and their combination on tumor compactness. HE-stained tumor sections at ×10 magnification show tumor compactness. Less compact areas are outlined in black, and their relative proportion was quantified. ***P < 0.001. (**f**) Immunofluorescence labelling of hypoxia-inducible factor 1 was used to evaluate the oxygen supply to the tumor. Scale bars denote 50 μm. Quantification of hypoxia areas was conducted to evaluate the oxygen supply across the groups. **P* < 0.05, and ****P* < 0.001.

**
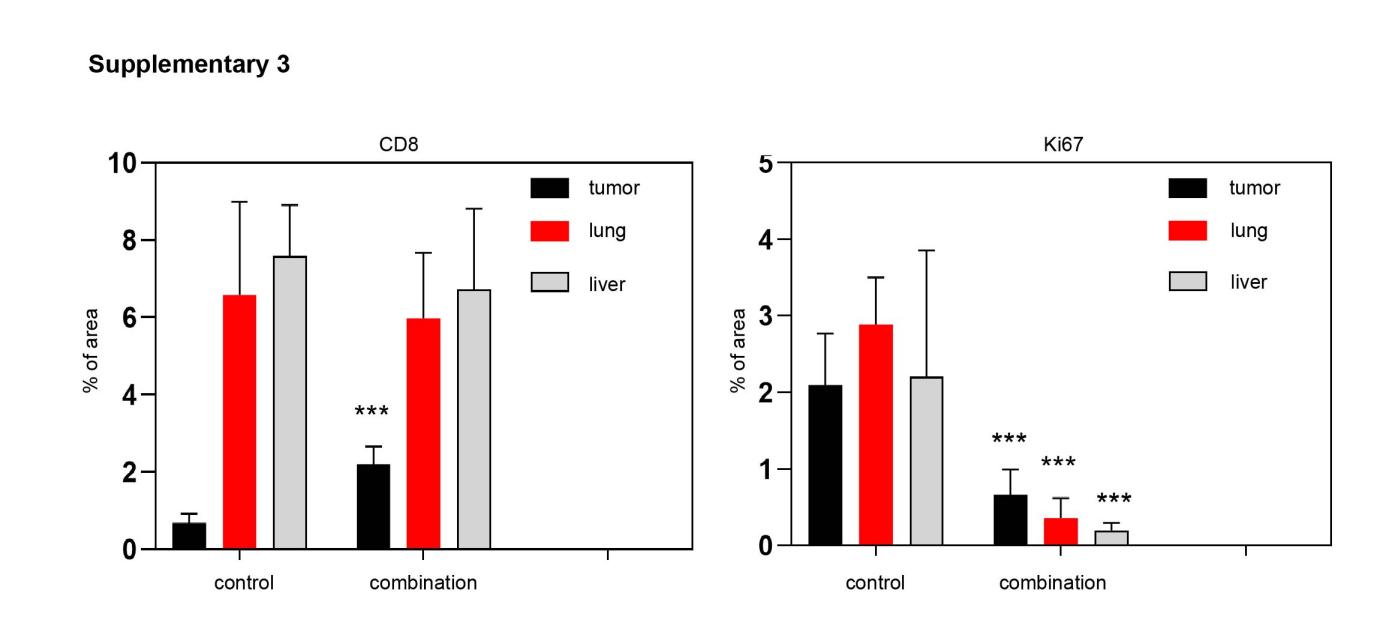
**

**Fig. S3. Quantification of histochemical results in control and neoadjuvant combination therapy groups.** (**a-b**) Immunohistochemical analysis and quantification of CD8^+^ T cell and Ki67 in metastasis foci of liver and lung, as well as primary tumor. The histochemical results of the two groups were quantified and compared. ***P < 0.001.
